# Political motivations for intra-European migration

**DOI:** 10.1177/0001699316659909

**Published:** 2016-08-07

**Authors:** Susanne Bygnes, Aurore Flipo

**Affiliations:** University of Bergen, Norway; Institut d’Etudes Politiques, Grenoble, France

**Keywords:** Exit, protest, Norway, Romania, Spain, political socialization

## Abstract

Motivations for migrating within the European Union have mainly been attributed to economic, career and lifestyle choices. This article suggests that political dissatisfaction is also an important motivator of recent intra-European migration. In our analysis of in-depth interviews with Romanian migrants in Spain and with Spanish migrants in Norway, we found a common emphasis on the political dimensions of their decision to migrate. In the interviews, the economic component of migration was often related to bad governance and negative perceptions of the state. The similarities of Spanish and Romanian migration narratives are especially striking because Spain and Romania represent substantially different migratory, political and economic contexts. However, migration is more obviously intertwined with conventional acts of political protest in the Spanish case. We suggest that differences in democratic contexts are pivotal in people’s reactions to and framing of their deep dissatisfaction with domestic politics, as found in many European countries today.

## Introduction

This article draws on cases from two of the most important intra-European mobility patterns in modern Europe, namely east–west and south–north migration. Thus far, extensive research has been conducted on the experiences of migrants from the eastern parts of Europe, who have left in great numbers for western Europe (e.g. Bleahu, 2004; Briggs and Dobre, 2014; Burrell, 2009). However, with a few notable exceptions (e.g. Bygnes, 2015; Triandafyllidou and Gropas, 2014; Jendrissek, 2016; Bygnes and Erdal, 2016), in-depth analyses of the recently re-established migration route from southern European countries are lacking. In this article, we analyse migration motivations and pay particular attention to the ways in which intra-European migrants from Romania and Spain articulate political dissatisfaction in their narratives. Although political dissatisfaction has long been studied in relation to emigration in autocratic contexts (e.g. see Colomer, 2000; Fleck and Hanssen, 2013), the importance of political discontent as a potential driver of emigration in democratic contexts has rarely been considered (for exceptions, see Hiskey et al., 2014; Lapshyna, 2014; Meardi, 2012; Triandafyllidou and Gropas, 2014). Therefore, when we analysed our material we were surprised to find a common emphasis on political motivations for migration across contrasting migration contexts. While Spanish migrants have come to northern European countries in the wake of an economic boom followed by a crisis, Romanians have left for western European countries to escape a post-communist era that has offered little economic betterment for ordinary citizens. Politically, post-crisis Spain has fostered social movements and widespread popular protest, while in Romania such reactions have until recently remained rare.

In this paper, we aim to raise awareness of the possible influence of political discontent on migration decision-making. We do this by exploring the political motivations woven into narratives provided by Spanish migrants in Norway and Romanian migrants in Spain. Previously, studies on motivations to migrate within Europe have focused more on pull factors such as economic and labour market opportunities in other parts of the continent, or on aspects of self-realization and the lure of consumer culture (e.g. see Hadler, 2006; McGhee and Trevena, 2012; Recchi and Favell, 2009). These are also among the main motivations found in interviews with highly skilled European professionals (notably, see Favell, 2008) and with lifestyle and retirement migrants (e.g. see Lundström, 2014). Although such factors are clearly important, recent research suggests that some intra-European migrants are also pushed by particularly deep dissatisfaction with their current location and a lack of future prospects (Triandafyllidou, 2006; Triandafyllidou and Gropas, 2014). However, until now the relationship between political discontent and migration in Europe has not been systematically explored. Therefore, in this article we draw on Albert O Hirschman’s (1970, 1978) concepts of ‘exit’ and ‘voice’, and social movement scholar Charles Tilly’s (2006) concept of ‘repertoires of contention’, to facilitate an exploration of (a) the embedding of political discontent in the narratives of intra-European migrants and (b) differences in the framing of political discontent as a potential driver of migration across migration contexts. Drawing on migrant narratives from two contrasting cases of intra-European migration allows us to speculate on the role of specific national and political contexts in the articulation of political dissatisfaction and migration narratives. We do not attempt to separate political and economic motives, but rather to illustrate how they are linked in migrants’ narratives, where economic turmoil is often attributed to a lack of democracy and vice versa.

## Analytical framework

People’s strategies in the face of perceived injustice are key themes in theories of social movements and collective action (Gamson et al., 1982; Snow and Benford, 1988). Tilly’s ‘repertoires of contention’ illustrate how the specific *tools of protest* available to a social movement are culturally constructed as ‘claim-making routines’ and vary according to political, social and historical context (2006: 35). Although emigration is rarely considered a form of protest by scholars of collective action, it has been analysed by migration scholars as a response to dissatisfaction in terms of Hirschman’s (1970) concepts of ‘exit’ and ‘voice’ (e.g. see Ådnanes, 2004; Meardi, 2012). Originally developed to categorize people’s alternatives when discontented with an organization, exit was initially understood by Hirschman (1970) to be the ‘passive’ and individual reaction, and voice as the ‘active’, public and politically oriented action. However, in his subsequent analyses of emigration from the German Democratic Republic, Hirschman (1993) suggested that ‘exit’ can interact with ‘voice’, and that the strategies reciprocally enhance each other. Hirschman’s concepts of exit and voice have since been drawn on by a range of scholars to examine emigration as an act of political protest. Most of these studies have focused on the impact of different diasporas on internal politics (e.g. see Kapur, 2010; Meseguer and Burgess, 2014), especially in the context of autocratic regimes (Colomer, 2000; Fleck and Hanssen, 2013).

Political participation ‘from the outside’ by emigrants has also been a key theme in research on political transnationalism (e.g. see Guarnizo et al., 2003; Moses, 2005; Waldinger and Fitzgerald, 2004; Østergaard-Nielsen, 2003). Research from the US context has found that migrants’ level of engagement in transnational political practices varies according to both educational level and country background, indicating that transnational political activities need prior political resources (Waldinger, 2015) and are certainly not ‘the refuge of marginalized or poorly educated immigrants’ (Guarnizo et al., 2003: 1238). Similarly, cross-country analyses in the European context find that the most highly educated intra-European migrants are the most likely to be politically active (Favell, 2010; Recchi and Favell, 2009).

Research on intra-European migrants’ political behaviour indicates considerable variations within the European context. For example, Ettore Recchi (2015) has found that Romanian citizens at home and abroad stand out as the least politically active of all Europeans. Romanians are less likely to engage in protests, sign petitions or vote than any other European citizens. Spanish citizens abroad, on the other hand, are described as both ‘the most radical’ and ‘the most likely to engage in protest’ of all European citizens abroad (Muxel, 2009: 177). These findings suggest that political socialization varies greatly across the European continent and tends to influence people’s political behaviour when they migrate (Recchi, 2015: 114). Furthermore, the analysis by Anne Muxel shows that intra-European migrants are more politically engaged than citizens who stay put (2009). Compared with the general population, EU movers tend to combine left-leaning economic attitudes with a liberal attitude to moral and cultural issues (Favell, 2010).

Research on the political behaviour of intra-European migrants also reveals an interesting paradox: while those who emigrate are more likely to label politics a matter of high interest, their actual *participation* in elections and other political activities is far lower than that of their compatriots (Favell, 2010; Muxel, 2009; Recchi, 2015). Although a lower turnout in national general elections may be attributed to institutional obstacles that limit the emigrant vote, it should be noted that intra-European movers also rank lower than ‘stayers’ on several other dimensions of politicization (Muxel, 2009). Therefore, based on previous research, we suggest that emigration is a possible course of action for politically engaged and dissatisfied individuals who feel that the ‘repertoires of contention’ at their disposal are insufficient for channelling their political discontent.

## Data and methodology

The data on Romanians in Spain (20 interviewees) and Spaniards in Norway (46 interviewees) drawn on in this article were collected separately for two projects with different starting points (see project information in biographical notes). The difference in sample sizes stems from this fact. Interviewees from both projects were adult women and men. We included participants with higher levels of education working in their professions or in jobs below their qualification levels, as well as people with lower levels of education. Despite the difference in sample sizes, there was significant diversity among the Romanian and Spanish participants in a number of characteristics.

The semi-structured biographical interviews of Romanians in Spain focused on school-to-work transitions and labour market integration, but also more broadly on the migration decision-making process and future prospects. The issue of political discontent as a driver of migration arose from an analysis of narratives about past experiences and future plans. The 20 Romanian migrants interviewed were recruited in the Valencian community between 2010 and 2011. The respondents were selected through personal contacts in Romania and Spain and through a Romanian cultural association, using snowball sampling as the main recruiting technique. The interviewees (12 females, eight males) were between 18 and 36 years of age and had been in Spain for an average period of approximately five years. The majority of the Romanians worked in manual occupations, but two worked as teachers, one worked in the cultural sector and one was a qualified nurse. Among the manual workers, four people held university degrees, including master’s degrees.

Biographical semi-structured interviews were conducted with 46 Spanish nationals (15 females, 31 males) who had arrived in Norway after the 2008 crisis hit and had resided there for an average of two years. They were aged between 22 and 60 years, but most were in their 30s and early 40s. Half of the Spanish interviewees worked in highly skilled jobs or were looking for work in this sector of the labour market and had completed a university degree equivalent to a master’s, and most had relevant work experience in Spain. The other half worked in jobs that did not require a university education. About half of these were deskilled; that is, they held higher education degrees but worked in manual jobs. We made contact with the Spanish interviewees through three Facebook networks and from fieldwork over the course of one month with a local non-governmental organization. We also drew on personal contacts in two large companies and snowball sampling as additional recruitment techniques. The interviews were conducted in Oslo, Bergen and Stavanger between March 2013 and September 2014, and focused broadly on the migrants’ lives and experiences before coming to Norway and after their arrival.

The common analysis of data from the two projects was developed by searching for recurring themes in the interview material. The stories and backgrounds of the 20 Romanians in Spain and 46 Spaniards in Norway were naturally highly diverse, but a common theme across the material was the relationship between political dissatisfaction and previous or future migration plans. The striking similarity of the grievances we found in our samples and the relative absence of this topic in the literature convinced us of the value of this enterprise. We bore in mind that our data were limited in scope and certainly not representative of all intra-European migrants. However, the exploration of two contrasting cases of recent intra-European migration allows us to conduct a pioneering in-depth analysis of political disenchantment in migration narratives across contexts, and to draw some analytical generalizations.

## Different contexts, similar grievances

Although migration and mobility have been important in Romania for many decades, the migratory route from Romania to Spain is quite recent. The most important waves of migration from Romania to Spain occurred between 2002 (when Spain abolished the visa obligation for Romanian citizens) and the 2008 crisis. In 2007, the accession of Romania to the EU allowed Romanians in Spain to acquire the status of European citizens and the right of residence, followed by the full opening of the labour market in 2009. However, despite the pre-2009 restrictions, various political measures aimed at facilitating the employment of Romanians were enforced to cater to the labour demands of Spain’s booming economy (Ribas-Mateos, 2004). The number of Romanian citizens in the 2011 Spanish census was over 750,000, about 14% of the total foreign population of Spain (INE, 2012).

The migration route from Spain to Norway is also a recent one. In the post-dictatorship years, the migration of Spanish nationals to other European countries was very limited. However, between 2008 and 2012 almost 700,000 left the country, more than from any other southern European country in the same period (González-Ferrer, 2013). A small proportion of these moved to Norway, where a once virtually non-existent Spanish population has grown to almost 5000 in the wake of the crisis (Statistics Norway, 2014).

Spain had experienced three decades of economic betterment followed by an economic boom, resulting in a relatively high standard of living and positive expectations for the future, before the crisis hit in 2008, whereas the prospects in Romania were much less bright. Except for an urban and well-qualified middle class minority, the overwhelming majority of Romanians suffered severely from the economic transition from socialism; in the 2000s, two-thirds of the population considered themselves poorer than before 1989 (Popescu et al., 2009). In the wake of the privatization process, inequality has increased, while democratic performance remains poor. According to the Barometrul de Opinie Publică (BOP, 2007), only 21% of Romanians were satisfied with the way their democracy worked, and 90% considered corruption ‘rather’ or ‘very’ widespread. For many years now, Romania has been at the bottom of the Transparency International Corruption Perceptions Index ranking for EU countries.

Furthermore, until very recently, people with grievances about the labour market or social justice issues have had a very limited ‘voice’ in the Romanian political arena, and the media rarely addresses topics linked with social inequalities (Popescu et al., 2009). Because of Romania’s socialist past, leftist parties have lacked the legitimacy needed to talk about social justice, leaving few possibilities for criticism of economic liberalism. Domestic politics has focused entirely on the transition towards the market economy and the EU (Cretu, 2014). Since 2012, however, the situation has changed. Following the partial privatization of the emergency services, significant protests were organized in Romania, combining anti-government protests with anti-austerity and anti-corruption demands (Volintiru, 2012). The discontent focused especially on the former prime minister, Victor Ponta, who was forced to resign with his government in November 2015.

By contrast, in the wake of the 2008 crisis, protest movements were quickly mobilized across Spain against political corruption, increasing social inequality and the effects of austerity measures (Campos Lima and Artiles, 2013). The economic collapse that followed a decade of economic boom created very abrupt changes in Spanish society. Unemployment rose to 26%, and soared to 56% for those under 25 (Eurostat, 2013). One of the social movement organizations established in the wake of the crisis was an initiative by expatriate Spaniards who had left after the crisis struck, *Marea Granate* (Maroon Wave). The movement’s manifesto emphasizes that the economic and social crisis has ‘forced’ them to ‘flee’ from Spain (www.mareagranate.org). The establishment of the better-known *Indignados* movement (a version of *Occupy*), the anti-corruption and anti-establishment party *Podemos* and social movement organizations such as *Marea Granate* can all be seen as reactions to the record low levels of trust in Spanish politicians captured in opinion polls in 2014; 80% of those polled rated the general political situation in Spain as ‘bad’ or ‘very bad’ (CIS, 2014).

## The articulation of economic and political motives for migration

To analyse people’s motives for migration, we draw on C Wright Mills’ (1940: 909) suggestion that a quest for ‘real motives’ over ‘mere rationalization’ does not make sense because ‘the only social items that lie deeper are other lingual forms’. We acknowledge that drawing on honourable categorizations such as that of the ‘political émigré’ plays a part in the symbolic boundary work of highly educated and/or politically engaged migrants (Bygnes, 2015). However, according to Mills (1940), this does not make the political motivations of the migrants in this study less ‘real’. The motives expressed in the interviews are analysed as ‘a basis of inference for a typal vocabulary of motives of a situated action’ because people’s motivational structures ‘are relative to societal frames’ (Mills, 1940: 911).

The Spaniards interviewed for this study came from all walks of life and had various levels of resources available to them in both Spain and Norway. The highly educated and often well-paid segment of migrants from Spain tended to emphasize that their decision to migrate had relatively little to do with economic factors, although the particularly favourable labour market opportunities and low unemployment rate in Norway (about 3%) were also factored into their decision narratives. Very few of the highly skilled interviewees reported having lost their jobs before leaving Spain. However, several participants expressed anxiety about the high unemployment rate in their home country at the time of their departure, for example Sebastian, a geologist employed in Norway’s petroleum sector, said that:

If the Spanish situation were completely different, maybe I would still be in Spain. I mean, there is a lot […] there is a crisis; there is a crisis because of corruption, and there is a high unemployment rate, and also, because I have a child. I mean, just thinking about [our child] is […] I was a little bit scared. You know, last year, I said, ‘OK, maybe what happens if I lose my job?’ […] So thinking about that, you know, it’s complicated. (Sebastian, 30, geologist)

On the other hand, among the Spanish interviewees who either worked in low-skilled occupations or were looking for manual work, it was much more common to highlight economic factors as central to their decision to migrate. In this group, most reported having lost their jobs in Spain or having worked in very precarious positions before making the decision to leave. Thus far, the differences between the narratives of the highly skilled and lower-skilled Spaniards in Norway are quite unsurprising; similar profiles are well documented in the research literature, indicating that class and educational level influence motivations for migrating within Europe (e.g. see Favell, 2008; Lundström, 2014). However, looking beyond these first impressions reveals a similarity between the narratives of the ‘highly skilled’ and the ‘workers’ that was far less expected, namely a common and deep dissatisfaction with the political situation in Spain.

The Romanian migrant interviewees also came from a range of socio-economic backgrounds and had different education levels. Almost all the interviewees focused on having left Romania for Spain in a period when Romania was facing economic hardship and high unemployment, while Spain was experiencing tremendous growth. The significant salary gap between Spain and Romania at the beginning of the 2000s also played a central role in the migration motivations reported. For the most highly skilled migrants, migration to Spain was a way to seek better opportunities and circumvent employment difficulties in the Romanian labour market. Amelia explained:

After he finished his studies in medicine, my boyfriend had to do a compulsory one-year internship in various services of the hospital, and then he was supposed to find a position but there was nothing. It was only supposed to be for a few months. I never thought we would actually live in Spain. (Amelia, 30, PhD)

Thus, many of the highly skilled migrants initially saw their migration as temporary, because they expected Romania’s economy to improve with the country’s accession to the EU in 2007. In addition to the salary gap, almost all the Romanian interviewees pointed to high unemployment, the lack of meritocracy in the labour market and the problem of corruption as reasons for leaving Romania or deciding not to return. Overall, economic considerations were stressed as core motivations for migration in all the Romanian narratives, differing somewhat from the general tendency of the migration motives reported by Spaniards in Norway. However, the economic component of the Romanian narratives of migration was in nearly all cases linked with a remarkable degree of distrust in the country’s politicians and institutions. The dissatisfaction with the situation in Romania was widely shared by migrants and seemed to increase during their stay in Spain, as more than half declared they did not wish to return permanently: ‘I don’t see how it’s ever going to change’, said Mircea (36, delivery driver). For a large number of the Spanish interviewees too, political dissatisfaction was expressed as a lack of faith in the political system, and contempt for corruption and the inability of political leaders to deal with debt, unemployment and economic difficulties in a way that benefited people. Economic reasons for leaving cited by several of the lower-skilled Spanish interviewees were also explicitly intertwined with political dissatisfaction.

Although our analysis is dedicated to exploring political motivations for migration, we should note that in a small number of the interviews, political motivations for migration were not mentioned at all. In such instances, the migrants tended to highlight work, career opportunities or family as the main motivations for migration. For example, Penelope, a 32-year-old domestic worker from the south of Spain, decided to follow her Norwegian employers when they moved back to Norway in 2010 because she needed the work. She estimated her chances of obtaining work in the south of Spain as close to zero and framed the choice of following her employers to Norway as her only option to provide for herself and her family. In contrast to the overwhelming majority of our research participants, she was eager to go back the moment she or her husband could obtain employment in Spain. However, the most common motivations in both the Spanish and the Romanian cases were different combinations of political and economic dissatisfaction, with an emphasis on a lack of faith in the future of the home society.

## Unwrapping the political dimension

Dissatisfaction with politics and society was often high on the agenda in our interviews with Spanish citizens in Norway. Although the extent to which informants explicitly related their motivation for leaving Spain to their dissatisfaction with the state of affairs in their home country varied somewhat, interviewees in both high- and low-skilled occupations insisted that politics in Spain was a disgrace. Javier, a university drop-out in low-skilled employment in Norway, most clearly expressed his exit as an act of political protest:

The way I see it, I am in Norway 100% for political reasons. [Those leaving Spain at the moment] leave as a consequence of terrible politics, and the mediocrity of politicians who are supposed to govern us, but are more concerned about debts than they are with people. (Javier, 28, kitchen assistant)

For Romanians, political dissatisfaction was often expressed as strong disappointment and exasperation with the everlasting ‘transition process’ that was expected to bring employment and welfare to the country. Political leaders and institutions were described as incapable of improving the country’s situation, and the impression that nothing appeared to be changing was accentuated by the constant promises of a better future that had been made since the regime change in 1989. The paradox is illustrated by the common saying ‘before we had money but the stores were empty; now they are full but our pockets are empty’. For example, Ciprian, who graduated from a school of economics in Bucharest, found no decent employment opportunities after graduation. Ciprian left for Spain for what he expected to be a short period, to save money to start his own life. Like many other graduates, he thought the situation in the country would improve during his time abroad, and that he would be able to go back after a few years. However, Ciprian had been in Spain for seven years and had lost faith in any chance of improvement back home:

It makes me sick when I realize that it’s getting worse instead of better. And it makes me so furious […] because that’s what put me here, that’s what I told you before. I can’t go back [to Romania] because of them. Because of the politicians and of what’s going on over there. Because of this anger, I prefer to stay here.

Among the Romanians interviewed in Spain, the most educated appeared to be the most politically aware, consistent with Muxel’s (2009) findings. Graduates assumed that their degrees would guarantee them some social mobility, even if it meant having to stay abroad for a while. They had high expectations for the country’s development, but had been severely disappointed. Some of them, such as Amelia, a 30-year-old PhD graduate, consider themselves part of a ‘sacrificed generation’:

I have a bit of hatred towards my own country, because it made me go through difficulties, and it never gave us any opportunities, the young people. […] It’s not worth it for me. It’s just a waste of time to think about it, about what is going on in Romania. So I have moved away, it’s just like I have erased the country from my memory.

The inability of the interviewees to envision a positive future for themselves was a common way of expressing their motivations to migrate from both Spain and Romania. The young and deskilled Spanish migrants, sometimes referred to as Spain’s ‘lost generation’, frequently mentioned the lack of a future: ‘I cannot create a life for myself in my own country. I have no opportunities. No future. I have nothing’ (Adela, 36); ‘I decided to leave, to jump the sinking ship in order to save my youth and my future’ (Javier, 28). The feeling is also common among the highly skilled and affluent. For example, Theodor works in the petroleum sector in Bergen and comes from a wealthy family in Spain. Nevertheless, he chose to leave Spain because, as he said, ‘I saw no future in Spain. I mean, of course my family has some means, so I could always have worked with them for one of the many companies we have there, but I didn’t want to’ (Theodor, 29).

In both the Spanish and the Romanian cases, many well-educated people talked about leaving because of an absence of meritocracy, which seemed to fuel their political discontent and distaste for the home country (e.g. see Rabikowska, 2010). However, for many Romanians, the economic crisis in Spain brought back the fear of downward mobility and a lack of future prospects at the centre of their initial decision to emigrate. Living in an indeterminate ‘in between’, many felt disoriented and adopted a wait-and-see strategy. Cristian, an unskilled construction worker, left Romania because of the ‘lack of a future’ he saw for himself there. He lost his job in Spain during the crisis, and with his unemployment benefits he barely had enough to survive: ‘For now, I live in the present. I can’t see anything better right now’. Going back to Romania does not seem like a better option: ‘[In Romania] wherever you go, whatever door you try to open, you have to bribe. […] In Romania nothing has changed, so for me nothing has changed either’. Initial disappointment with Romania was often combined with disappointment with the situation in Spain and Europe in general. Manuela, a PhD candidate and English teacher, confessed: ‘I didn’t think it would be that hard here too. Everywhere you have to fight to make a life for yourself’. Another issue at the heart of most of the migrants’ dissatisfaction with politics was corruption, a concern they shared with most of their compatriots (e.g. see BOP, 2007; CIS, 2014) and which extended across all social categories. For example, Diana, a 34-year-old Spanish shop assistant in Oslo, linked the motivation behind migration to corruption, noting that so many people were leaving because of the terrible situation in Spain, and ‘it is all related to politics and the politics is horrible now; it’s corrupt’ (Diana, 34). For Romanian migrants as well, corruption was held responsible for the poor performance of the economy and the inefficiency of public services. Mirela, a 37-year-old health care assistant, suggested that ‘they don’t know what to do with the money from the European Union, how to invest it […]. For people to have better lives […]. That’s because of politicians. They are the guilty ones’.

The deep political distrust and anger at corruption are directed at political and business elites in general, referred to as ‘the caste’ in Spanish anti-corruption rhetoric or ‘the dogs’ in the case of Romania (see Briggs and Dobre, 2014: 84). Spaniard Alfredo, an unemployed control operator in Bergen, explained that corruption was sadly indispensable to survive as a politician in Spain, and in fact party affiliation made no difference: ‘the right wing party [or the left wing party], all of that’s a lie, all of that’s so we’ll believe that democracy exists in Spain, but in reality they are the same people’ (Alfredo, 47). Anti-corruption has, since 2014, been a main element in the successful mobilization of *Podemos* against the two mainstream parties that Alfredo alluded to. For Anita, a 31-year-old cultural mediator, Romanian politics could also be easily summarized: ‘If I say “corruption”, in one word I have told you everything’.

Despite different political histories and contexts of migration, both Romanian and Spanish migrants focused on strikingly similar issues when expressing their political discontent: corruption, lack of trust in the political system and a poor outlook for betterment in the future in their home countries. Both Romanian and Spanish migration narratives revealed a sense of having been ‘let down’ or ‘sacrificed’ by their governments. Previous research across contexts has shown that people’s attitudes towards the future are at the core of migration decisions; emigration is more likely to occur when future prospects are bleak, while political engagement, on the other hand, is linked with hope (e.g. see Dhillon and Yousef, 2009; Ådnanes, 2004). However, different repertoires of contention are not mutually exclusive, and the same individuals can choose to migrate and to protest or otherwise voice their discontent both before and after migrating. In the following section, we show that this is certainly the case for the migrants from Spain.

## Transnational engagement

In the Spanish narratives, we find examples of both active political protest followed by migration and of continuous protest and political engagement from new transnational vantage points. Importantly, as Sidney Verba (1962: 23) has argued, if an ordinary citizen perceives ‘government policy as being far outside of his sphere of influence’, she or he will be less likely to attempt to influence the government. Political socialization and ‘the extent to which people perceive themselves as competent to influence the government’ are ‘closely related to the extent of democracy in that country’.

Several Spanish interviewees mentioned taking part in street demonstrations against the political and social status quo before deciding to leave. For example, Javier (28) and Chimo (27) left Spain after having actively ‘voiced’ their dissatisfaction by participating in several protest events organized by the *Indignados* movement and other local initiatives. Javier connected his departure from Spain with people leaving public squares in the wake of the *Indignados* protests that took place all over Spain in 2011:

The root cause of my leaving was that during the 15 May demonstrations everyone came out to camp and protest on squares in different cities against the incapacity of the government to resolve the problems of the crisis. And after I participated in this movement, I decided to leave [Spain] … to jump ship, because the ship was sinking. (Javier, 28)

Javier frames his participation in the *Indignados* protests as part of a politicization process, which eventually played a key role in his decision to leave Spain. Among the Spanish interviewees who actively continued to voice their protests after arriving in Norway, Javier also participated in activities organized by the recently established *Podemos* branch in Norway. Another example of participation in transnational political protest was Helena’s descriptions of the spring and summer of 2011, when she first arrived in Norway. Helena had a highly skilled job in Bergen, and she talked about her participation in several demonstrations in support of the *Indignados*:

Like two years ago, there was a huge demonstration in Spain against the politicians, complaining about the whole situation in Spain, then there was also a demonstration in Bergen, and then I just went there […] not knowing anyone, just because I agreed with the demonstration. I think Norwegians thought we were crazy, like 20 Spanish people shouting and demonstrating in Bergen. It was really weird […] and then we held another demonstration in June because in Spain they actually had tents in the squares and they were sleeping there, protesting, and then in Barcelona the police started to get aggressive with the people, and then in June there was another demonstration [in Bergen] to protest about this aggression. (Helena, 30)

During the interview, deskilled Adela (36) talked about participating in a *Podemos* meeting in Oslo. Like a handful of those interviewed after the establishment of the party in 2014, Adela mentioned that she planned to vote for *Podemos*. Spanish carpenter Ramón (50) made it clear that he was economically very well off before the crisis hit and did not mention having been previously engaged in political protest. Despite this, he said he wanted to use his transnational vote for *Podemos* as a protest against ‘those who steal’, referring to ‘the caste’ of corrupt politicians and officials frequently mentioned in the political rhetoric of *Podemos*. Thus, in the Spanish case, voice and exit sometimes overlapped, and either added a political dimension to migration motivations framed primarily as economic or career oriented, or articulated even more clearly political dissatisfaction already expressed in the narratives.

Witnessing the *Indignados* movement in Spain, although many Romanian migrants feared that sudden political changes might place them in jeopardy by changing the conditions of their stay, several supported the grievances of the social movements: ‘When the government is doing something bad, Spaniards don’t remain seated […]. They go in the streets and fight for themselves’, said Catalina, a 26-year-old Romanian barmaid interviewed in 2011, deploring the lack of popular protest in her country. At the time the interviews were conducted, Romanian democracy was defined as ‘apathetic’ by most interviewees. However, in recent years, diverse expressions of popular discontent have appeared both inside and outside the country. In the Romanian expatriate communities of Madrid and London and in all the main cities of Romania, protests were organized in the run-up to the 2014 Romanian presidential elections. Prime Minister Ponta was accused of limiting the number of polling stations abroad to prevent transnational votes from becoming too influential. The unexpected victory of the centre-right candidate Klaus Iohannis, presented as the ‘anti-corruption candidate’, was attributed by analysts to the influence of the expatriate vote. Indeed, political parties in Romania have actively reached out to a transnational audience (Østergaard-Nielsen and Ciornei, 2013), and expatriate participation in the 2014 presidential elections turned out to be slightly higher than usual (Vilcu, 2014). Additionally, some of the most politicized and educated Romanian migrants, such as Amelia (30), reported that they had directed their political engagement towards their host country: ‘I am more interested in Spanish politics now […] because this is where I live’. However, recent studies on the incorporation of Romanian migrants in local politics indicate low civic participation and a lack of ‘mobilization resources’ in Spain (Ciornei, 2016).

## Conclusion

Our analysis showed that intra-European migrants’ decisions to leave and unwillingness to return to their home countries had a political component that was frequently raised and heavily emphasized in the material at hand. These tendencies are in line with recent research suggesting that political motivations seem to be part of current intra-European migration (e.g. see Meardi, 2012; Triandafyllidou and Gropas, 2014). The narratives that we have analysed in two relatively different European contexts add to previous research by showing that motives such as personal economic prospects, career considerations and family life are often interwoven with a distaste for the political situation at home.

Consistent with previous findings (e.g. see Guarnizo et al., 2003; Recchi, 2015), our analysis indicates that although the articulation of economic and political motives is widespread, they are framed somewhat differently depending on the person’s educational and occupational status, but even more so on national background. Among highly skilled Spanish migrants working at the high end of the Norwegian labour market, several declared that political dissatisfaction was one of their main motives for leaving, even before the interview commenced. This was also the case for some of the deskilled and very politically conscious Spaniards in Norway. On the other hand, among the Romanians in Spain and the Spaniards with low-skilled and unskilled work in Norway, it was more common initially to frame their motivations for leaving as purely economic and to elaborate on political dissatisfaction during the interview, especially when asked about their plans for return. The analysis thus showed how political discontent sometimes appeared as a ‘hidden motivation’ in the material; while economic motives were cited as the main reason for leaving, people blamed politicians for the failing economy and for their gloomy prospects. They particularly emphasized the lack of democratic accountability of political elites and that they took insufficient responsibility for social justice. By drawing on in-depth qualitative interviews, we have shown that dissatisfaction with politics may factor into the decision to migrate, even among those whose migration decisions could be classified as ‘economic’ in survey research. Our findings support recent research on migration drivers, suggesting that separating ‘political’ and ‘economic’ migration fails to take into account the multifaceted catalogue of motives that migrants relate to in practice (e.g. see de Haas, 2011).

Dealing with key differences in the material, we found that Spanish migrants tended to describe their own exit as a more explicitly political act than did the Romanian migrants. In the Spanish narratives, voice and exit not uncommonly went hand in hand, while a ‘silent exit’ was more prevalent among the Romanian migrants. In the Romanian case, emigration was often reported as the only available response to dissatisfaction. Although transnational Romanian migrants have been claimed in the past to be important contributors to social change (Potot, 2006), the relative lack of active social movements in Romania when the fieldwork was conducted may partly explain why dissatisfaction has been expressed far less through conventional means of protest than in the Spanish case.

Following Meardi (2012; see also Triandafyllidou and Gropas, 2014), it may be argued that migrants are ‘voting with their feet’, but we note that most people do not migrate, thus complicating the view of political dissatisfaction as a driver for migration. However, our in-depth qualitative interviews lend support to recent findings that European movers are more politically engaged than stayers (Recchi, 2015). The anti-elite discourse often drawn on in our interviews may provide a possible answer to the question of why mobile Europeans on the whole are more politically aware but much less willing to go to the polls than citizens who stay put (Recchi, 2015). Therefore, based on our analysis and the previous literature, we suggest that some of those who migrate within Europe at the moment are politically engaged and dissatisfied Europeans who feel that the ‘repertoires of contention’ at their disposal are not sufficient for channelling their political discontent. Furthermore, the Romanian and Spanish cases we have drawn on in our analysis indicate that whether people choose to voice their protests in addition to leaving when dissatisfied with their current locations must be understood in connection with varying opportunities for protest and differences in political socialization across European regions and countries.

## References

[bibr1-0001699316659909] Bleahu A (2004) Romanian migration to Spain. Motivation, networks and strategies In: Pop D (ed.) New Patterns of Labour Migration in Central and Eastern Europe. Cluj Napoca: AMM, pp.20–35.

[bibr2-0001699316659909] BOP – Barometrul de Opinie Publică (2007) Bădescu, Comşa, Sandu et Stănculescu, *Barometrul Opinie Publică 1998–2007*. Bucharest: Soros Foundation Romania.

[bibr3-0001699316659909] Briggs D Dobre D (2014) Culture and Immigration in Context: An Ethnography of Romanian Migrant Workers in London. Basingstoke: Palgrave Macmillan.

[bibr4-0001699316659909] Burrell K (2009) Polish Migration to the UK in the ‘New’ European Union: After 2004. Burlington, VT: Ashgate.

[bibr5-0001699316659909] Bygnes S (2015) Are they leaving because of the crisis? The sociological significance of *anomie* as a motivation for migration. Sociology. Available at: http://soc.sagepub.com/content/early/2015/06/29/0038038515589300.abstract

[bibr55-0001699316659909] Bygnes S Erdal MB (2016) Liquid migration, grounded lives: considerations about future mobility and settlement among Polish and Spanish migrants in Norway. Available at: http://dx.doi.org/10.1080/1369183X.2016.1211004.

[bibr6-0001699316659909] Campos Lima MDP Artiles A (2013) Youth voice(s) in EU countries and social movements in southern Europe. European Review of Labour and Research 19(3): 345–364.

[bibr7-0001699316659909] Ciornei I (2016) European mobility and local political incorporation: The case of British and Romanian residents in Spain. Migration Studies 4(1): 38–58.

[bibr8-0001699316659909] CIS (2014) Distribuciones Marginales, Barómetro de Abril. Madrid: Centro De Investigaciones Sociologicas (CIS).

[bibr9-0001699316659909] Colomer J (2000) Exit, voice, and hostility in Cuba. International Migration Review 34(2): 423–442.

[bibr10-0001699316659909] Cretu G (2014) Avatars de la nouvelle démocratie en Roumanie. Outre-Terre 4(41): 225–233.

[bibr11-0001699316659909] De Haas H (2011) The determinants of international migration: Conceptualising policy, origin and destination effects. DEMIG Project Paper 2 Oxford: International Migration Institute, University of Oxford.

[bibr12-0001699316659909] Dhillon N Yousef T (eds) (2009) Generation in Waiting: The Unfulfilled Promise of Young People in the Middle East. Washington, DC: Brookings Institution Press.

[bibr13-0001699316659909] Eurostat (2013) Euro area unemployment rate at 12.2%. Available at: http://epp.eurostat.ec.europa.eu/cache/ITY_PUBLIC/3–31102013-BP/EN/3–31102013-BP-EN.pdf (accessed 28 May 2014).

[bibr14-0001699316659909] Favell A (2008) Eurostars and Eurocities: Free Movement and Mobility in an Integrating Europe. Oxford: Blackwell.

[bibr15-0001699316659909] Favell A (2010) European identity and European citizenship in three “Eurocities”: A sociological approach to the European Union. Politique européenne 30(1): 187–224.

[bibr16-0001699316659909] Fleck R Hanssen A (2013) When voice fails: Potential exit as a constraint on government quality. International Review of Law & Economics 35: 26–41.

[bibr17-0001699316659909] Gamson W Fireman B Rytina S (1982) Encounters with an Unjust Authority. Homewood, IL: The Dorsey Press.

[bibr18-0001699316659909] González-Ferrer A (2013) La nueva emigración española. Lo que sabemos y lo que no. *Zoom Politico* 2013/18. Madrid: Laboratorio De Alternativas.

[bibr19-0001699316659909] Guarnizo LE Portes A Haller W (2003) Assimilation and transnationalism: Determinants of transnational political action among contemporary migrations. American Journal of Sociology 108(6): 1211–1248.

[bibr20-0001699316659909] Hadler M (2006) Intentions to migrate within the European Union: A challenge for simple economic macro-level explanations. European Societies 8(1): 111–140.

[bibr21-0001699316659909] Hirschman A (1970) Exit, Voice, and Loyalty: Responses to Decline in Firms, Organizations, and States. Cambridge, MA: Harvard University Press.

[bibr22-0001699316659909] Hirschman A (1978) Exit, voice and the state. World Politics 31(1): 90–107.

[bibr23-0001699316659909] Hirschman A (1993) Exit, voice and the fate of the German Democratic Republic: An essay in conceptual history. World Politics 45(2): 173–202.

[bibr24-0001699316659909] Hiskey J Montalvo J Orcés D (2014) Democracy, governance, and emigration intentions in Latin America and the Caribbean. Studies in Comparative International Development 49(1): 89–111.

[bibr25-0001699316659909] INE (2012) Demographic census of 2011. Instituto Nacional de Estadisticas. Available at: http://ine.es/censos2011_datos/cen11_datos_inicio.htm (accessed 15 March 2015).

[bibr56-0001699316659909] Jendrissek D (2016) Building a future in times of crisis. Young highly qualified migrants in the UK’. Journal of Contemporary European Studies. doi:10.1080/14782804.2015.1117965.

[bibr26-0001699316659909] Kapur D (2010) Diaspora, Development, and Democracy: The Domestic Impact of International Migration from India. Princeton, NJ: Princeton University Press.

[bibr27-0001699316659909] Lapshyna I (2014) Corruption as a driver of migration aspirations: The case of Ukraine. Economics & Sociology 7(4): 113–127.

[bibr28-0001699316659909] Lundström C (2014) White Migrations: Gender, Whiteness and Privilege in Transnational Migration. London: Palgrave Macmillan.

[bibr29-0001699316659909] McGhee DS Trevena P (2012) Dignity, happiness and being able to live a ‘normal life’ in the UK—An examination of post-accession Polish migrants’ transnational autobiographical fields. Social Identities 18(6): 711–727.

[bibr31-0001699316659909] Meardi G (2012) Social Failures of EU Enlargement: A Case of Workers Voting with Their Feet (Routledge Research in Employment Relations) New York: Routledge.

[bibr32-0001699316659909] Meseguer C Burgess K (2014) International migration and home country politics. Studies in Comparative International Development 49(1): 1–12.

[bibr33-0001699316659909] Mills CW (1940) Situated actions and vocabularies of motive. American Sociological Review 5(6): 904–913.

[bibr34-0001699316659909] Moses JW (2005) Exit, vote and sovereignty: Migration, states and globalization. Review of International Political Economy 12(1): 53–77.

[bibr35-0001699316659909] Muxel A (2009) EU movers and politics: Towards a fully-fledged European citizenship? In: Recchi E Favell A (eds) Pioneers of European Integration: Citizenship and Mobility in the EU. Cheltenham and Northampton, MA: Edward Elgar Publishing, pp.156–178.

[bibr36-0001699316659909] Popescu M Hatieganu V Muntean A (2009) Social inequality and why it matters for the economic and democratic development of Europe and its citizens. Post-Communist Central and Eastern Europe in comparative perspective. EUREQUAL Report for Romania Oxford: University of Oxford.

[bibr37-0001699316659909] Potot S (2006) Les migrants transnationaux acteurs de la transition post-communiste In: Krasteva A Todorov A (eds) Modernisation, Démocratisation, Européanisation: La Bulgarie et la Roumanie Comparées. Sofia: Nouvelle Université Bulgare, pp.259–269.

[bibr38-0001699316659909] Rabikowska M (2010) Negotiation of normality and identity among migrants from Eastern Europe to the United Kingdom after 2004. Social Identities 16(3): 285–296.

[bibr39-0001699316659909] Recchi E (2015) Mobile Europe. London: Palgrave Macmillan.

[bibr40-0001699316659909] Recchi E Favell A (eds) (2009) Pioneers of European Integration. Citizenship and Mobility in the EU. Cheltenham and Northampton, MA: Edward Elgar Publishing.

[bibr41-0001699316659909] Ribas-Mateos N (2004) How can we understand immigration in southern Europe? Journal of Ethnic and Migration Studies 30(6): 1045–1063.

[bibr42-0001699316659909] Snow D Benford R (1988) Ideology, frame resonance, and participant mobilization. International Social Movement Research 1: 197–218.

[bibr43-0001699316659909] Statistics Norway (2014) Befolkningsstatistikk for 2014. Available at: http://ssb.no/befolkning/statistikker/innvbef/aar/2014–04–24?fane=tabell&sort=nummer&tabell=176208 (accessed 20 June 2014).

[bibr44-0001699316659909] Tilly C (2006) Regimes and Repertoires. Chicago, IL: The University of Chicago Press.

[bibr45-0001699316659909] Triandafyllidou A (2006) Contemporary Polish Migration in Europe: Complex Patterns of Movement and Settlement. Lewiston, NY: Edwin Mellen Press.

[bibr46-0001699316659909] Triandafyllidou A Gropas R (2014) “Voting with their feet”: Highly skilled emigrants from southern Europe. American Behavioral Scientist 58(12): 1614–1633.

[bibr47-0001699316659909] Verba S (1962) Political participation and strategies of influence: A comparative study. Acta Sociologica 6(1): 22–42.

[bibr48-0001699316659909] Vilcu DA (2014) Romanian diaspora: The 2014 presidential elections as positive community practice. Journal of Community Positive Practices 14(4): 113–127.

[bibr49-0001699316659909] Volintiru C (2012) Romania’s recent protests have become a social movement calling for the dignity of the people in the face of an unaccountable government. LSE Blogs EUROPP. Available at: http://blogs.lse.ac.uk/europpblog (accessed 16 May 2015).

[bibr50-0001699316659909] Waldinger R (2015) The Cross-Border Connection: Immigrants, Emigrants, and their Homelands. Cambridge, MA: Harvard University Press.

[bibr51-0001699316659909] Waldinger R Fitzgerald D (2004) Transnationalism in question. American Journal of Sociology 109(5): 1177–1195.

[bibr52-0001699316659909] Østergaard-Nielsen E (2003) The politics of migrants’ transnational political practices. International Migration Review 37(3): 760–786.

[bibr53-0001699316659909] Østergaard-Nielsen E Ciornei I (2013) Political parties and the transnational mobilization of the emigrant vote In: Midwest Political Science Association Conference, Chicago, IL, 11–18 April 2013.

[bibr54-0001699316659909] Ådnanes M (2004) Exit and/or voice? Youth and post-communist citizenship in Bulgaria. Political Psychology 25(5): 795–815.

